# A fuzzy-augmented deep learning pipeline for multilingual natural language processing from handwritten script: a Tamil case study

**DOI:** 10.3389/frai.2026.1859443

**Published:** 2026-10-14

**Authors:** Harsha Kumar A, B. Aarthi, K. Kumaran, G. Saranya, Preetham Venkatram C

**Affiliations:** School of Computer Science and Engineering, Vellore Institute of Technology, Chennai, India

**Keywords:** deep learning, machine learning and artificial intelligence, multilingual translation, natural language processing, neuro-fuzzy systems, pattern recognition, sequence-to-sequence models, Tamil Handwritten Character Recognition (THCR)

## Abstract

Multilingual natural language processing (NLP) from handwritten script is an open artificial intelligence (AI) challenge requiring advances in both pattern recognition and sequence generation. Existing end-to-end pipelines often fail to model the structural uncertainty in complex scripts, particularly the decorative stroke variability in Dravidian writing systems, before passing representations to downstream NLP models. This omission can result in error propagation from the recognition stage into translation. This paper proposes a Tamil Handwritten Character Recognition and Translation (THCRT) framework, a fuzzy-augmented deep learning NLP pipeline that addresses this gap by coupling a Biparametric Exponential Membership Function-based Fuzzy Inference System (BEMFFIS) with a deep convolutional recognition network and a multilingual transformer for translation. The fuzzy inference component models stroke-boundary uncertainty through Decorative Stroke Analysis (DSA) using the Coefficient of Variation (CoV). BEMFFIS employs a coupled-parameter Gaussian membership function, which reduces the hyperparameter search space to a single degree of freedom. This produces normalized representations that improve downstream NLP performance. Hierarchical segmentation of line, word, and character is achieved via Connected Polar Functional Component Analysis (CPFCA). Recognition is performed by a Global Averaged Excitation-based Deep Convolutional Neural Network with Swish activation (GAE-DCSSNN), and multilingual translation is executed by a multilingual Bernoulli Distribution-based Auto-Regressive Transformer (mBBDART) built on the mBART backbone with stochastic token-pruning augmentation. The pipeline is evaluated on Tamil handwriting using two publicly available datasets: the Tamil Offline Handwritten Word Recognition (TOHWR) dataset and the Tamil Handwritten Character Corpus Mendeley Data (THCCMD) dataset. Translation quality is assessed on a 1,200-sample parallel reference set annotated by two certified bilingual translators with Cohen's κ = 0.91, reflecting the word-level and short-phrase scope of the TOHWR corpus. The THCRT framework achieves a recognition accuracy of 98.23%, a Character Error Rate (CER) of 1.12%, and a Bilingual Evaluation Understudy (BLEU) score of 0.438 on the annotated evaluation set, outperforming the baseline architectures on both datasets and demonstrating a 1.79 percentage-point accuracy gain attributed to the BEMFFIS stroke-uncertainty module.

## 1 Introduction

Multilingual handwritten script recognition remains an open challenge due to the complexity and diversity of varied regional scripts (Jabde et al., 2025). In morphologically complex and low-resource languages, this structural variability introduces uncertainty that propagates through recognition into downstream NLP tasks such as machine translation. Tamil Handwritten Character Recognition (THCR) is a well-characterized instance of this challenge: Tamil is one of the oldest Dravidian languages, and the inherent cursive nature of its script along with numerous diacritical marks introduces complexities for character recognition models (Devendiran et al., 2024). Applications include document digitisation (Chauhan et al., 2024), automated document analysis (Shaffi and Hajamohideen, 2021b), and cross-language communication (Jabde et al., 2025).

Several limitations characterize the existing literature. Established recognition frameworks often neglect decorative stroke and stroke-width variation analysis, reducing accuracy on complex scripts. Conventional translation approaches rely on word-to-word mapping and overlook contextual relationships, which can produce grammatical errors in the output (Bamotra and Uppala, 2023). Many segmentation techniques focus on line and character separation while omitting word-level segmentation, potentially causing semantic misinterpretation in the final output. Deep learning models for Tamil, such as MobileNet-based systems (Vinotheni and Pandian, 2023), frequently overlook geometric and stroke-peak variations, which can yield false predictions for structurally similar characters. Collectively, these limitations highlight a need for principled mechanisms for modeling stroke-boundary uncertainty before handwritten representations are passed to deep learning classifiers and sequence-to-sequence models.

The proposed THCRT framework addresses these limitations through four targeted contributions. BEMFFIS models decorative stroke uncertainty via CoV extraction prior to deep learning classification, addressing the first limitation. Dependency Parsing (DP) establishes syntactic structure before mBBDART generates grammatically coherent multilingual translations, addressing the second. CPFCA performs hierarchical line, word, and character segmentation using a Polar transformation that handles curved Dravidian baselines, addressing the third. GAE-DCSSNN integrates geometric, structural, and frequency-based features with a channel excitation mechanism and Swish activation, addressing the fourth.

The THCRT framework achieves a recognition accuracy of 98.23% and a CER of 1.12% on the TOHWR dataset, and 97.61% accuracy on the THCCMD dataset. For translation, a BLEU of 0.438 and a Metric for Evaluation of Translation with Explicit ORdering (METEOR) of 0.512 are achieved on a 1,200-sample expert-annotated parallel set. An ablation study indicates that removing BEMFFIS reduces accuracy by 1.79 percentage points and increases the CER by 1.49 ×, confirming its contribution to pipeline performance.

The paper is organized as follows: Section 2 surveys recent literature. Section 3 describes the proposed THCRT methodology. Section 4 details the experimental setup. Section 5 presents the ablation study. Section 6 reports results and discussion. Section 7 concludes the work.

Table 1 lists all abbreviations used in this paper.

**Table 1:** List of Abbreviations.

Abbreviation	Full Form	Abbreviation	Full Form
AI	Artificial Intelligence	IoU	Intersection over Union
BEMF	Biparametric Exponential Membership Function	LBP	Local Binary Pattern
BEMFFIS	BEMF-based Fuzzy Inference System	LSTM	Long Short-Term Memory
BiGRU	Bidirectional Gated Recurrent Unit	METEOR	Metric for Evaluation of Translation with Explicit ORdering
BLEU	Bilingual Evaluation Understudy	MF	Median Filter
CCA	Connected Component Analysis	mBART	Multilingual BART
CER	Character Error Rate	mBBDART	Multilingual BD-based Auto-Regressive Transformer
CLAHE	Contrast-Limited Adaptive Histogram Equalization	mBERT	Multilingual BERT
CNN	Convolutional Neural Network	MLT	Multilingual Language Translation
CoV	Coefficient of Variation	NLP	Natural Language Processing
CPFCA	Connected Polar Functional Component Analysis	OCR	Optical Character Recognition
DCNN	Deep Convolutional Neural Network	PP	Projection Profile
DP	Dependency Parsing	RLSA	Run-Length Smoothing Algorithm
DSA	Decorative Stroke Analysis	ROUGE	Recall-Oriented Understudy for Gisting Evaluation
FFT	Fast Fourier Transform	SR	Segmentation Rate
FIS	Fuzzy Inference System	SiLU	Sigmoid Linear Unit
FNR	False Negative Rate	ST	Segmentation Time
FSL	Few-Shot Learning	THCR	Tamil Handwritten Character Recognition
GAE	Global Averaged Excitation	THCCMD	Tamil Handwritten Character Corpus Mendeley Data
GAE-DCSSNN	GAE-based Deep CNN with Swish activation	THCRT	Tamil Handwritten Character Recognition and Translation
GHT	Guo-Hall Thinning	TOHWR	Tamil Offline Handwritten Word Recognition
HOG	Histogram of Oriented Gradients	WER	Word Error Rate
HT	Hough Transform	WSO	Water Strider Optimization
ZM	Zernike Moments	BD	Bernoulli Distribution

## 2 Literature survey

Recent advances in deep learning, NLP, and fuzzy inference have influenced AI-based handwritten character recognition and multilingual translation. Vinotheni and Pandian (2023) established an end-to-end Tamil handwritten document recognition model using deep learning, where noise removal alongside line and character segmentation preceded feature extraction via MobileNet and classification using a Bidirectional Gated Recurrent Unit (BiGRU) optimized by the Water Strider Optimization (WSO) algorithm. The model was limited to a single language and did not incorporate fuzzy uncertainty modeling. Kavitha and Srimathi (2022) assessed offline THCR via a Convolutional Neural Network (CNN) classifier achieving high accuracy, but without decorative stroke analysis. Shaffi and Hajamohideen (2021a) explored Few-Shot Learning (FSL) using Siamese-CNN with minimal samples, though the approach assumed pre-segmented inputs rather than handling raw documents.

Bamotra and Uppala (2023) introduced an OCR pipeline integrated with LSTM sequence translation where image distortion and noise caused reading inaccuracies. Kavitha et al. (2023) validated THCR using the uTHCD dataset and pre-trained VGG-16, focusing on isolated character evaluation rather than word-level recognition.

Regarding heritage preservation, Ezhilarasi and Uma Maheswari (2025) introduced SignaryNet for recognizing heritage inscription palaeography using Deep Neural Networks, noting that its deeper architecture requires additional computational resources compared to classic models. Maheswari et al. (2024) developed an intelligent segmentation system for ancient Tamil palm-leaf manuscripts using augmented projection profiles and CNNs, mapping all characters into 125 distinct classes to reduce neural network complexity. Murugan and Visalakshi (2024) advanced recognition of ancient Tamil inscriptions using the hybrid DR-LIFT framework, though the model remains limited to stone inscriptions. Anbalagan et al. (2026) proposed TamilSTARNet, a tri-phase segmentation and attention-based recognition architecture for handwritten Tamil documents, reporting state-of-the-art accuracy on character recognition benchmarks.

In the domain of attention-based models, Rastogi et al. (2026) introduced SCRAT-Net, a deep learning architecture for handwritten word recognition that demonstrates strong generalization across diverse scripts, providing a recent and competitive baseline for the present work.

In forensic document analysis, Dansena et al. (2022) evaluated pen ink discrimination by combining statistical global features with local motif textural patterns. To optimize such ink analysis, Dansena et al. (2020) assessed seven different color models and identified YCbCr as the most effective representation for differentiating pen inks. Furthermore, to address the data scarcity in training deep learning models for these forensic tasks, Dansena et al. (2021) introduced a framework for generating synthetic handwritten word alteration datasets using English character samples. Oyshe et al. (2025) integrated an AdH-CNN for Bangla Sign Language. While effective for isolated classification, the system lacked a semantic sequence model, restricting it to static label prediction rather than generative sequence output, a limitation the proposed mBBDART addresses through subword-level, dependency-informed lexical translation.

In the broader Indic text processing literature, CNNs have been applied to handwritten Tamil scripts (Krithiga et al., 2025), Feature Pyramid Networks have been utilized for handwritten Bengali equations (Das et al., 2024), and CNNs for handwritten character recognition coupled with LSTM networks for language translation have been developed for Tulu (Prathwini et al., 2024). Although Fateh et al. (2024) used attention mechanisms for multilingual numeral recognition, this approach does not account for the decorative stroke complexities unique to Tamil. Studies by Saqib et al. (2022) and Zanwar et al. (2023) improve foundational English character recognition using deep learning and ensembled machine learning techniques, yet neither extends these methods to sequential text or downstream NLP tasks like translation.

Table 2 summarizes the existing works. The surveyed literature indicates that the integration of a fuzzy inference mechanism for structural uncertainty modeling prior to the deep learning recognition and multilingual translation stages remains largely unexplored. Addressing this gap in NLP architecture design is the primary motivation for the proposed THCRT framework.

**Table 2:** Summary of existing works.

Authors	Objective	Method	Result	Drawback
Vinotheni and Pandian (2023)	Tamil handwritten recognition	BiGRU and WSO	Precise recognition.	Single-language; no fuzzy uncertainty modeling.
Bamotra and Uppala (2023)	OCR with translation	LSTM	Translation completed.	Vulnerable to OCR misreads and image noise.
Kavitha and Srimathi (2022)	Offline THCR	CNN	High accuracy.	No decorative stroke analysis.
Shaffi and Hajamohideen (2021a)	FSL for THCR	Siamese-CNN	Precise THCR.	No line/word segmentation.
Kavitha et al. (2023)	THCR model	CNN and VGG-16	High accuracy.	Word-level recognition not achieved.
Ezhilarasi and Uma Maheswari (2025)	Heritage inscription recognition	DNN and SignaryNet	Strong recognition.	Deeper architecture increases computational demands.
Dansena et al. (2022)	Pen ink discrimination	Motif texture analysis	Precise recognition.	Focuses strictly on ink properties, not structural analysis.
Maheswari et al. (2024)	Character segmentation	Augmented HPP and CNN	Characters identified.	Requires mapping to a reduced set of 125 classes.
Murugan and Visalakshi (2024)	Tamil inscription recognition	TextSnake and CNN	Inscriptions identified.	Limited to stone inscriptions.
Anbalagan et al. (2026)	Tamil character recognition	TamilSTARNet (tri-phase + attention)	State-of-the-art accuracy.	Focuses solely on recognition; lacks translation.
Rastogi et al. (2026)	Handwritten word recognition	SCRAT-Net (attention)	Strong generalization.	Not Tamil-specific; no fuzzy pre-processing.
Dansena et al. (2021)	Word alteration detection	Deep learning	Words detected.	Limited to English datasets and ink analysis.
Dansena et al. (2020)	Pen ink determination	RGB, YCbCr, and ML	Ink variations identified.	Evaluates color models, omitting structural geometry.
Oyshe et al. (2025)	Sign language recognition	AdH-CNN	Precise static recognition.	No sequence model; static labels only.

## 3 Proposed THCRT methodology

The THCRT framework integrates fuzzy inference, deep convolutional recognition, and transformer-based multilingual translation into a unified end-to-end system. The pipeline processes a Tamil handwritten image through seven sequential stages: input collection, pre-processing, hierarchical segmentation, decorative stroke analysis, feature extraction and recognition, grammatical pattern analysis, and multilingual translation. A key design element is the introduction of a fuzzy uncertainty gate, BEMFFIS, between segmentation and feature extraction. This gate normalizes stroke-width variability before any pattern classification occurs, ensuring that the downstream deep learning components receive structurally consistent representations regardless of inter-writer variability. Figure 1 depicts the THCRT architecture.

**Figure 1:**
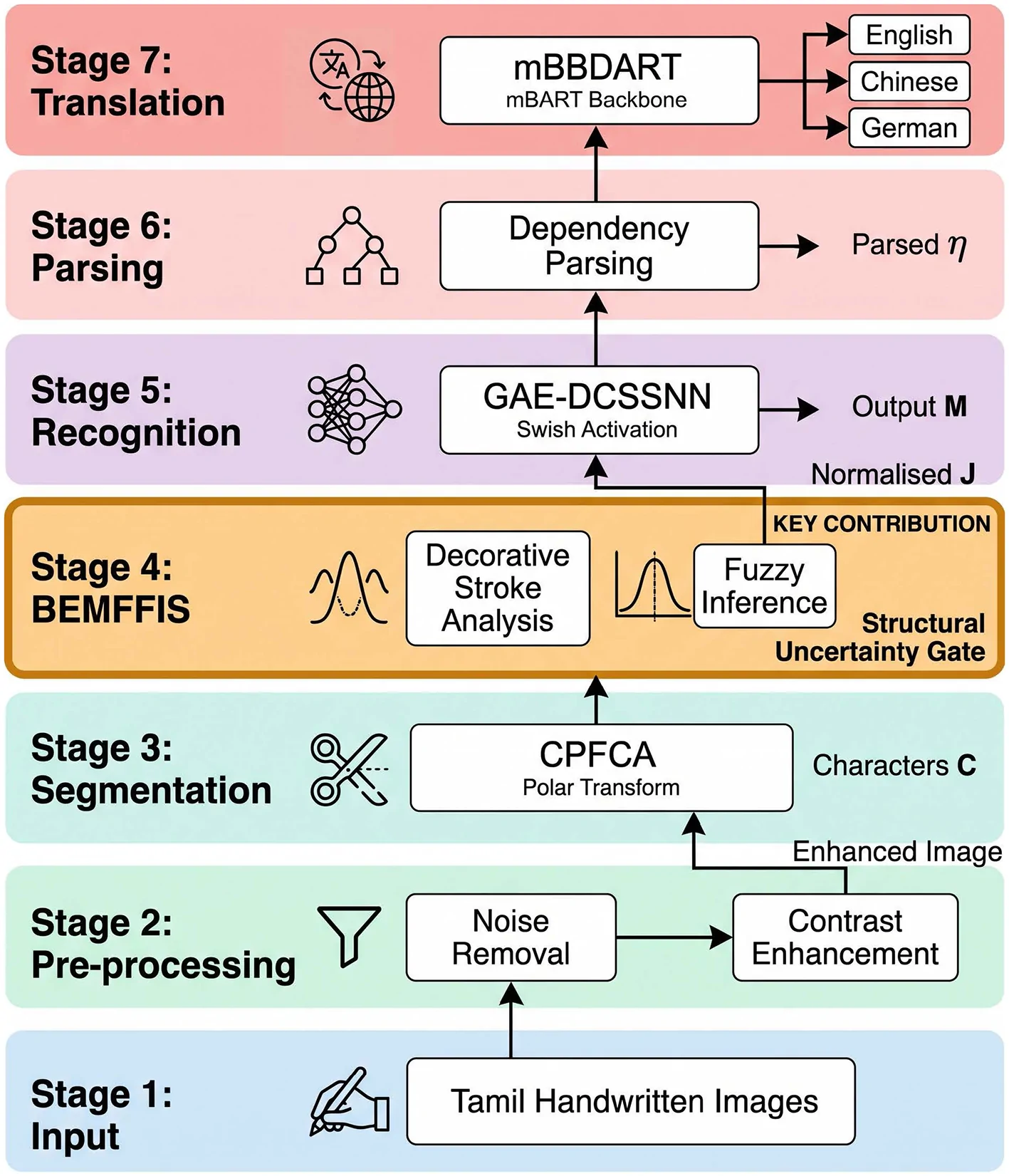
Architecture of the proposed THCRT framework. Data flow proceeds left to right through seven stages: input image collection, pre-processing, CPFCA segmentation, BEMFFIS decorative stroke analysis, GAE-DCSSNN recognition, Dependency Parsing, and mBBDART multilingual translation. The BEMFFIS block is highlighted as the structural uncertainty gate, intercepting the pipeline between segmentation and feature extraction to ensure all downstream components receive stroke-normalized representations.

Table 3 summarizes the mathematical notation used throughout this section.

**Table 3:** Summary of mathematical notation.

Symbol	Description	Symbol	Description
*T*	Input Tamil handwritten image set	$\tilde{T}$	Noise-removed image
$\ddot{T}$	Contrast-enhanced image	*K*	Polar-domain image
*C*	Segmented character region	*q*	Coefficient of Variation
$\bar{\chi }$	Mean stroke width	δ	Stroke width standard deviation
ϵ	Total pixel count in *C*	ℜ	Fuzzy rule set
*Z*	BEMF membership degree	Ĉ	Fuzzified character data
*J*	DSA score (defuzzified)	*F*	Stroke-detected image
*Q*	Extracted feature set	*E*	Combined classifier input {*J, F, Q*}
*H*	Convolutional feature map	β	Global average-pooled descriptor
ε	Channel excitation vector	$\tilde{H}$	Excitation-scaled feature map
*S*	Fully connected feature vector	*M*	Recognition output
η	Dependency parse graph	*L*	Token embedding matrix
*D*	Multilingual translation output	*	Spatial convolution
*A*	Swish-activated hidden vector	*W*_*out*_	Output classification matrix
⊙	Element-wise multiplication	σ(·)	Sigmoid function
φ	Ordered 8-neighbor binary sequence	ŵ	Decoded Tamil word string

### 3.1 Input image

The THCRT framework begins with the collection of Tamil Handwritten Images (THI), denoted *T* = {*T*_1_, *T*_2_, …, *T*_*f*_}, where *T*_*f*_ is the *f*^th^ collected image. A data quality assurance step described in Section 6.8 verifies benchmark ground truth before training and evaluation.

### 3.2 Image pre-processing

The validated image *T* is pre-processed via two sequential steps.

#### 3.2.1 Noise removal

Salt-and-pepper noise is suppressed using a Median Filter (MF), which replaces each pixel with the median intensity over a local neighborhood window *W*:


$$\begin{array}{l} \tilde{T}\left(x,y\right)={\mathrm{median}}_{\left(x^{\prime },y^{\prime }\right)\in W}\left\{T\left(x^{\prime },y^{\prime }\right)\right\} \end{array}$$ (1)

#### 3.2.2 Contrast enhancement

Contrast-Limited Adaptive Histogram Equalization (CLAHE) is applied to $\tilde{T}$. The image is divided into non-overlapping tiles of size *n*_*t*_ × *n*_*t*_, and each tile's histogram is clipped at limit *C*_*L*_:


$$\begin{array}{l} H_{c}\left(p\right)=\min\left(H\left(p\right),C_{L}\right),\;H\left(p\right)=\underset{\left(x,y\right)\in \text{tile}}{\sum }1\left[\tilde{T}\left(x,y\right)=p\right] \end{array}$$ (2)

The contrast-enhanced output $\ddot{T}$ is obtained via the cumulative distribution function CDF_*c*_ of the clipped histogram:


$$\begin{array}{l} \ddot{T}\left(x,y\right)=\left\lfloor \frac{{\text{CDF}}_{c}\left(\tilde{T}\left(x,y\right)\right)-{\text{CDF}}_{c,\min}}{n_{\text{tile}}-{\text{CDF}}_{c,\min}}\cdot \left(L-1\right)\right\rfloor \end{array}$$ (3)

### 3.3 Hierarchical segmentation (CPFCA)

Hierarchical segmentation of $\ddot{T}$ is performed using CPFCA. Standard Connected Component Analysis (CCA) in Cartesian coordinates often fails to map the curved baselines of Dravidian scripts, so a Polar transformation is applied first. The baseline center (*g*″, *h*″) is estimated as the centroid of the foreground pixels of each detected text line, computed via horizontal projection profile analysis. The polar coordinates (*m*, α) of each pixel (*g, h*) are:


$$\begin{array}{l} m=\sqrt{{\left(g-g^{\prime \prime }\right)}^{2}+{\left(h-h^{\prime \prime }\right)}^{2}},\;\alpha =\arctan\left(\frac{h-h^{\prime \prime }}{g-g^{\prime \prime }}\right) \end{array}$$ (4)

The polar-domain image *K* is:


$$\begin{array}{l} K\left(m,\alpha \right)=\ddot{T}\left(g^{\prime \prime }+m\cos\alpha ,h^{\prime \prime }+m\sin\alpha \right) \end{array}$$ (5)

CCA applied to *K* identifies connected components. Character regions *C* are recovered in Cartesian coordinates as:


$$\begin{array}{l} C=\left\{\left(m\cos\alpha +g^{\prime \prime },m\sin\alpha +h^{\prime \prime }\right)\right\}\cdot K \end{array}$$ (6)

### 3.4 Decorative stroke analysis (BEMFFIS)

The stroke-width variability within each segmented character *C* is analyzed to identify decorative elements before thinning. A Fuzzy Inference System (FIS) is chosen because it can represent vague stroke-boundary conditions through membership functions and fuzzy rules, which deterministic thresholding may not capture. The Biparametric Exponential Membership Function (BEMF) is selected over standard triangular, trapezoidal, or Gaussian functions because it reduces the tuning search to a single degree of freedom, reducing the risk of overfitting the membership parameters on a small validation subset while maintaining adaptability to inter-writer variability.

#### 3.4.1 CoV extraction


$$\begin{array}{l} \bar{\chi }=\frac{1}{\epsilon }\sum \chi \left(C\right),\;\delta =\sqrt{\frac{1}{\epsilon }\sum {\left(\chi -\bar{\chi }\right)}^{2}},\;q=\frac{\delta }{\bar{\chi }}\times 100 \end{array}$$ (7)

#### 3.4.2 Fuzzy rules

Based on *q*, characters are categorized as:


$$\begin{array}{l} ℜ=\{\begin{array}{cc} \text{if }q<30\% & \text{then }G^{1}\text{(well-formed script)}\\ \text{if }q\geq 30\% & \text{then }G^{2}\text{(decorative stroke)} \end{array} \end{array}$$ (8)

The 30% threshold was determined empirically on a held-out validation subset from the TOHWR training partition. Under *G*^1^, the stroke widths are already consistent, and the BEMF output *J* is passed through with minimal normalization by setting *t*_2_ → 0, which reduces the membership function to near-identity. Under *G*^2^, the full BEMF normalization is applied with learned *t*_1_ and *t*_2_ to suppress decorative width variance before downstream feature extraction.

#### 3.4.3 BEMF

The membership degree is:


$$\begin{array}{l} Z=\exp\left(-\left[{\left(\chi -t_{1}\right)}^{2}+t_{2}\cdot \chi^{2}\right]\right) \end{array}$$ (9)

where χ is the stroke-width at each foreground pixel, which is twice the Euclidean distance transform value, and *t*_1_, *t*_2_ ≥ 0 are tunable parameters. Completing the square gives $Z=h\cdot \exp\left(-\left(1+t_{2}\right){\left[\chi -t_{1}/\left(1+t_{2}\right)\right]}^{2}\right)$, where *h* cancels under centroid defuzzification. BEMF is therefore a Gaussian membership function whose center and precision are coupled through *t*_2_, reducing tuning to one degree of freedom. The Gaussian rows in Table 4 use an unconstrained Gaussian with independently tuned center *c* and width σ.

**Table 4:** BEMFFIS membership function comparison, averaged across TOHWR and THCCMD.

Membership function	Fuzz. (ms)	Defuzz. (ms)	Acc. (%)	CER (%)
Proposed BEMF	1.75	2.06	97.920	1.330
Gaussian	2.29	2.77	97.005	1.740
Triangular	1.48	1.65	94.975	2.725
Trapezoidal	1.91	2.16	95.650	2.340
Singleton	0.87	0.99	93.455	3.535

#### 3.4.4 Fuzzification and defuzzification

Each foreground pixel is independently fuzzified and defuzzified to produce the stroke-normalized output image *J*:


$$\begin{array}{l} {Ĉ}_{i}=C_{i}⊙Z_{i}\left(ℜ\right),\;J_{i}={Ĉ}_{i} \end{array}$$ (10)

The output *J* retains the spatial dimensions of *C* and is passed directly to the thinning stage.

Algorithm 1 formalizes the BEMFFIS procedure.

Algorithm 1 BEMFFIS procedure. 
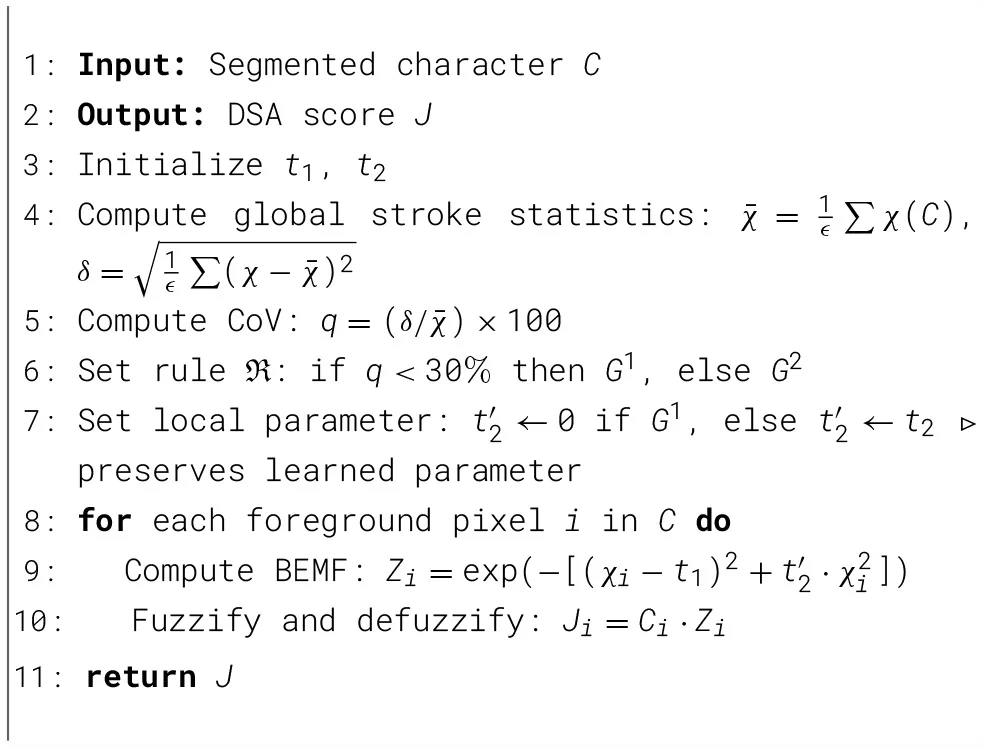

### 3.5 Feature extraction and recognition (GAE-DCSSNN)

#### 3.5.1 Stroke extraction

Guo-Hall Thinning (GHT) is applied to *J* to produce the stroke-detected image *F*. Let φ = (*P*_2_, *P*_3_, …, *P*_9_) denote the ordered binary values of the 8-connected neighbors of each foreground pixel, arranged clockwise. The transition count *w* is the number of 0 → 1 transitions in the circular sequence (*P*_2_, *P*_3_, …, *P*_9_, *P*_2_), and γ is the count of non-zero neighbors:


$$\begin{array}{l} w=\overset{9}{\underset{k=2}{\sum }}\left(1-P_{k}\right)\cdot P_{k+1},\;P_{10}\equiv P_{2},\;\gamma =\overset{9}{\underset{k=2}{\sum }}P_{k} \end{array}$$ (11)

A foreground pixel is retained in *F* if and only if 2 ≤ γ ≤ 6 and *w* = 1.

#### 3.5.2 Feature extraction

Features extracted from *F* and collectively denoted *Q* comprise Fast Fourier Transform (FFT)-based features such as magnitude spectrum, phase spectrum, and low- and high-frequency components; geometrical features including aspect ratio, stroke thickness, shape, length, angle, Zernike Moments (ZM), Histogram of Oriented Gradients (HOG), and intensity; and structural features like endpoint and intersection counts, Gabor features, Local Binary Pattern (LBP), and edge maps.

#### 3.5.3 GAE-DCSSNN

The combined input *E* = {*J, F, Q*} is passed to the GAE-DCSSNN classifier. The spatial inputs *J* and *F* are stacked as image channels and processed by the convolutional body, while the non-spatial feature vector *Q* bypasses the convolutional layers and is concatenated with the pooled representation at the first fully connected layer. Standard DCNNs discard inter-channel relationships through plain global average pooling and suffer from information loss for negative inputs under ReLU. GAE-DCSSNN addresses both by replacing global average pooling with a two-layer channel excitation network and replacing ReLU with Swish activation (Ramachandran et al., 2017). Figure 2 shows the classifier structure.

**Figure 2:**
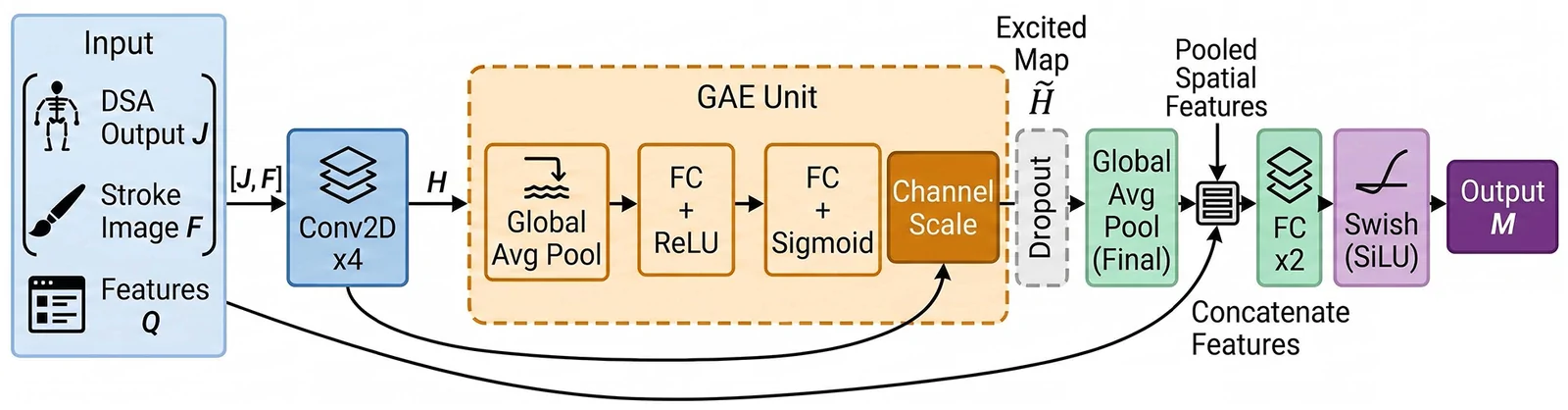
Structure of the GAE-DCSSNN classifier. The GAE unit is a two-layer bottleneck, FC-ReLU-FC-Sigmoid, applied after global average pooling. It produces a channel-wise excitation vector that re-weights feature maps before the fully connected head. The SS in the model name denotes Swish, or SiLU, activation used throughout the convolutional body.

The spatial feature map is computed from the spatial inputs only: *H* = ([*J, F*]*k) + *l*, where [*J, F*] denotes channel-wise stacking. The GAE block computes the channel descriptor $\beta =\frac{1}{cd}\underset{i,j}{\sum }H\left(i,j\right)$ and excites it via:


$$\begin{array}{l} \varepsilon =\sigma \left(W_{2}\cdot \mathrm{ReLU}\left(W_{1}\cdot \beta \right)\right),\;\tilde{H}=H⊙\varepsilon \end{array}$$ (12)

where $W_{1}\in {\mathbb{R}}^{c/r\times c}$, $W_{2}\in {\mathbb{R}}^{c\times c/r}$, *r* = 16. The pooled spatial representation is concatenated with the non-spatial feature vector *Q* and passed through the fully connected layer: $S=\left[\frac{1}{cd}\underset{i,j}{\sum }\tilde{H}\left(i,j\right),Q\right]\cdot W_{fc}+b_{fc}$. Swish activation (Ramachandran et al., 2017) is applied throughout the convolutional body and to the hidden representation:


$$\begin{array}{l} A=S\cdot \sigma \left(\beta_{s}\cdot S\right)=S\cdot \frac{1}{1+\exp\left(-\beta_{s}\cdot S\right)} \end{array}$$ (13)

where β_*s*_ is a learnable scalar initialized to 1. When β_*s*_ = 1 this is standard Swish, also known as SiLU. A final linear projection maps the activated features to class logits:


$$\begin{array}{l} M=A\cdot W_{out}+b_{out} \end{array}$$ (14)

where $W_{out}\in {\mathbb{R}}^{d\times N_{c}}$, *d* is the hidden dimension, and *N*_*c*_ is the number of classes, specifically 13,294 for TOHWR and 247 for THCCMD. The cross-entropy loss function applies softmax to *M* internally during training. Algorithm 2 formalizes the GAE-DCSSNN procedure.

Algorithm 2 GAE-DCSSNN procedure. 
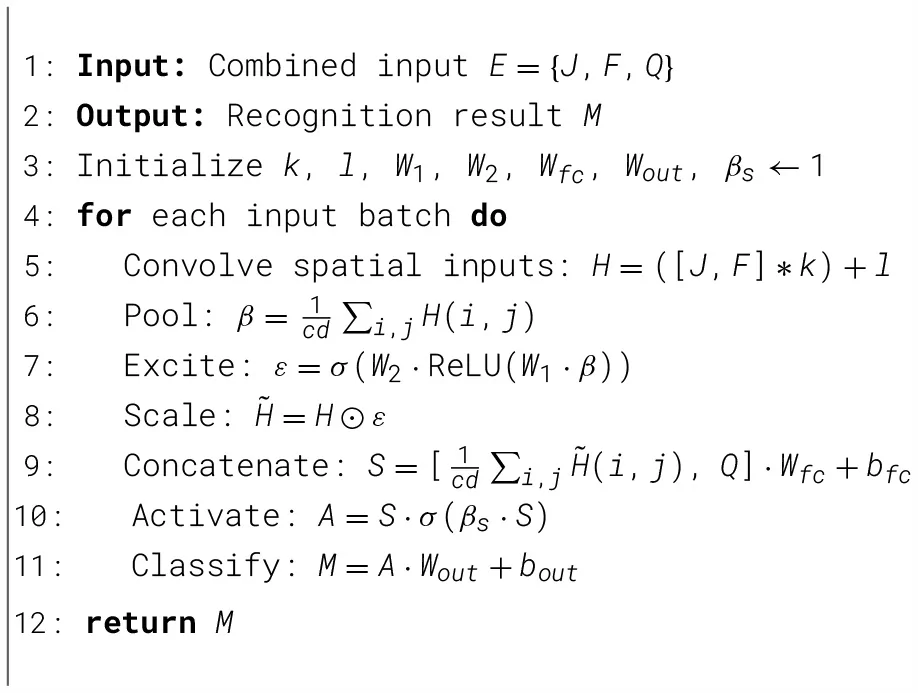

### 3.6 Grammatical pattern analysis

The recognition output *M* is decoded to its corresponding Tamil word string ŵ via the class-label dictionary. The string ŵ is segmented into subword units (*m*_1_, *m*_2_, …, *m*_*k*_) using the same mBART SentencePiece tokenizer applied in Section 3.7, so that *m*_*i*_ ≡ *u*_*i*_; no separate morphological analyser is used. Because Tamil is an agglutinative language in which a root combines with one or more grammatical suffixes, and because Tamil dependency treebanks at a scale sufficient to train robust statistical parsers are not widely available, the dependency structure η is constructed using a fixed rule rather than a learned parser. The first subword *m*_1_ is designated the head, and each subsequent subword is treated as a direct dependent of *m*_1_, reflecting the root-plus-affix pattern of Tamil morphology:


$$\begin{array}{l} \eta =\left\{\left(m_{1},m_{j},\rho \right)|j\in \left\{2,\ldots ,k\right\},\rho \in P\right\} \end{array}$$ (15)

where η is the dependency graph, *P* is the set of grammatical relation types, and ρ denotes the grammatical link between head subword *m*_1_ and dependent subword *m*_*j*_. For single-subword words, where *k* = 1 and which account for approximately 4% of TOHWR word types, η is empty and *m*_1_ is treated as an unmodified root with no dependents. This flat, head-centered structure requires no supervision or training data beyond the translation objective described in Section 3.7, and provides syntactic and morphological structure that informs grammatically coherent translation.

### 3.7 Multilingual language translation (mBBDART)

Multilingual Language Translation is performed by mBBDART, which augments the mBART encoder-decoder backbone (Liu et al., 2020) with a BD-based stochastic token-pruning scheme. The decoded word string ŵ is tokenized into subword tokens (*u*_1_, …, *u*_*N*_) by the mBART SentencePiece tokenizer, and each token is mapped to a continuous representation via the pre-trained mBART embedding layer, defined as *L*_*i*_ = Embed(*u*_*i*_). The dependency structure η is incorporated by adding a learned role embedding *R*_*i*_ ∈ {*R*_head_, *R*_dep_} to each token representation, updating it as *L*_*i*_ ← *L*_*i*_ + *R*_*i*_, marking whether *u*_*i*_ occupies the head or dependent role assigned by η. The parameters *R*_head_ and *R*_dep_ are learned end-to-end during mBBDART fine-tuning alongside the translation objective, without requiring separate supervision. A binary mask *s*_*i*_ is sampled per token dimension from a Bernoulli distribution parameterised by a learned score:


$$\begin{array}{l} s_{i}\sim \text{Bernoulli}\left(\sigma \left(w_{p}^{⊤}L_{i}\right)\right),\;x_{i}=s_{i}\cdot L_{i} \end{array}$$ (16)

Tokens with *s*_*i*_ = 0 are suppressed. This acts as structured dropout that focuses the encoder on semantically dense embeddings. During inference, all masks are set to *s*_*i*_ = 1 to disable token pruning, thereby ensuring deterministic translation output. The encoder produces contextual representation *I* = Encoder(*x*_1_, …, *x*_*N*_), and the decoder auto-regressively generates the translation *D* = Decoder(*I, u*_1_, …, *u*_*N*_). The THCRT pipeline achieves multilingual output through the mBART pre-training corpus, supporting 50 languages including English, Chinese, German, and Spanish. While the recognition component is Tamil-specific, extending CPFCA and BEMFFIS to additional Dravidian scripts such as Telugu and Kannada constitutes a planned future direction.

#### 3.7.1 Parallel evaluation set

A parallel reference set was constructed from the TOHWR held-out test partition. Two certified Tamil-English bilingual translators, working independently and without access to each other's annotations, translated 1,200 uniformly sampled test items into English. Inter-annotator agreement was Cohen's κ = 0.91; the 9% of items on which the two annotators disagreed were adjudicated by a third independent annotator. The resulting reference set, including individual annotations prior to final adjudication, is available via the repository listed in the Data availability statement. The TOHWR dataset comprises individual handwritten Tamil words, meaning each item corresponds to exactly one word token. BLEU, METEOR, and Recall-Oriented Understudy for Gisting Evaluation (ROUGE) are computed against these 1,200 annotated pairs using the sacrebleu toolkit. The BLEU score of 0.438 reflects the inherent difficulty of translating isolated handwritten words without sentence-level context. Generalization to long-form text would require evaluation on a corpus such as FLORES or Samanantar, constituting a distinct future direction.

## 4 Experimental setup

### 4.1 Hardware and software environment

The THCRT framework is implemented in Python 3.9. The GAE-DCSSNN is trained on an NVIDIA GPU with 12 GB GDDR6 memory. mBBDART fine-tuning is performed on Google Colab Pro with an NVIDIA A100 featuring 40 GB HBM2 to accommodate the memory requirements of the mBART backbone. The development system uses an Intel Core i9 processor at 3.20 GHz, Windows 10 64-bit, and 16 GB system RAM.

### 4.2 Dataset description

#### 4.2.1 TOHWR

The TOHWR dataset contains 103,518 handwritten Tamil word images spanning 13,294 distinct word types, partitioned into 75,736 training, 11,598 validation, and 16,184 test samples. Each item contains exactly one word, resulting in a single token per item. The test partition is used for all reported recognition and translation metrics. Both training and test samples are drawn from disjoint contributor groups, forming a writer-independent split, to prevent style memorisation.

#### 4.2.2 THCCMD

The THCCMD dataset contains 24,700 images across 247 Tamil character classes, equating to 100 samples per class, with 19,760 training and 4,940 test samples under a writer-independent 80/20 split. Dataset links are provided in the Data availability statement.

### 4.3 Evaluation metrics

Recognition performance is evaluated using accuracy, precision, recall, F-measure, which is the harmonic mean of precision and recall, CER, and False Negative Rate, calculated as 1 − Recall. CER is Levenshtein distance at Unicode codepoint level divided by reference length. On TOHWR, items are single words with a mean length of 9.8 codepoints. A misrecognised word may share codepoints with its prediction, so the CER of 1.12% is lower than the item error rate, 100%−98.23% = 1.77%. Word Error Rate (WER) equals the item error rate for this dataset because each item contains exactly one word token. All F-measures are the harmonic mean of reported precision and recall. Segmentation performance is evaluated using Intersection over Union (IoU), Segmentation Rate (SR), and Segmentation Time (ST). Translation performance is evaluated using BLEU, METEOR, and ROUGE on the 1,200-sample parallel annotation set. BLEU is computed on TOHWR only, as translation is not applicable to THCCMD character recognition.

### 4.4 Parameter settings

Table 5 details the parameter specifications for GAE-DCSSNN and mBBDART. Hyperparameters were selected by grid search optimizing validation CER for GAE-DCSSNN and validation BLEU for mBBDART.

**Table 5:** Parameter specifications for **(a)** GAE-DCSSNN and **(b)** mBBDART.

Parameter	Value
(a) GAE-DCSSNN	
Batch size	64
Epochs	50
Learning rate	0.001
Optimiser	Adam
Activation function	Swish (β_*s*_ = 1, learnable)
Loss function	Cross-entropy
Dropout rate	0.4
Kernel size	4 × 4
Weight initialization	He initialization
Regularization	L2
Convolutional layers	4
Dropout layers	2
Flatten layer	1
Fully connected layers	2
Output layer	1 (Softmax, *N*_*c*_ classes)
Training time per epoch	342 s^†^
(b) mBBDART	
Architecture	Encoder-decoder (mBART backbone)
Encoder / decoder layers	12 / 12
Hidden Size	1,024
Feed-forward width	4,096
Attention heads	16
Embedding dimension	1,024
Vocabulary size	250,000
Dropout rate	0.3
Label smoothing	0.1
Learning rate	5 × 10^−5^
Optimiser	AdamW
Warmup steps	500
LR scheduler	Cosine decay
Batch size	32
Maximum sequence length	512^*^
Gradient clipping	1.0
Loss function	Cross-entropy

^*^Permissive upper bound aligned with mBART defaults; TOHWR entries average 2 to 4 subword tokens, with a mean of 3.2.

^†^Pre-processing, including segmentation, BEMFFIS, and feature extraction, was performed offline prior to training. The reported training time covers only the CNN forward and backward pass on pre-computed feature tensors.

## 5 Ablation study

### 5.1 Pipeline module ablation

Table 6 evaluates the contribution of BEMFFIS and CPFCA to downstream NLP performance. Three configurations are compared against the full THCRT pipeline.

**Table 6:** Pipeline module ablation, averaged across TOHWR and THCCMD.

Pipeline configuration	Acc. (%)	CER (%)
Full THCRT pipeline	97.920	1.330
Without BEMFFIS (raw *C* to GAE-DCSSNN)	96.130	1.980
Gaussian FIS instead of BEMFFIS	97.005	1.740
CCA instead of CPFCA (no Polar transform)	96.965	1.650

Removing BEMFFIS reduces accuracy by 1.79 percentage points and increases CER from 1.330% to 1.980%, a factor of 1.49 ×, indicating that stroke-uncertainty normalization prior to deep learning classification improves performance. Replacing BEMFFIS with a Gaussian FIS recovers partial performance, achieving 97.005% accuracy and a CER of 1.740%, suggesting that the fuzzy preprocessing step is beneficial and that the BEMF formulation provides additional gain. Replacing CPFCA with CCA reduces accuracy to 96.965% and raises CER to 1.650%, showing that curved-baseline handling via the Polar transformation contributes to overall performance. Figure 3 shows the per-dataset breakdown.

**Figure 3:**
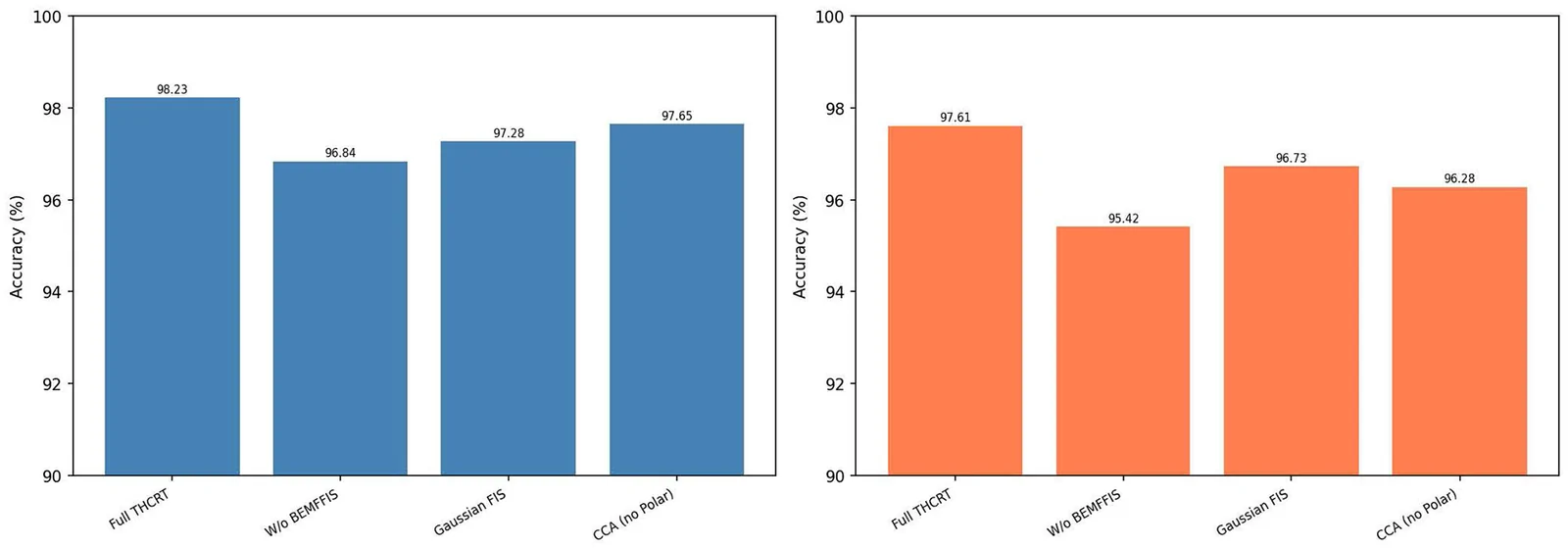
Pipeline module ablation per dataset. **Left**: TOHWR accuracy, **Right**: THCCMD accuracy. The averages across TOHWR and THCCMD correspond to the values reported in Table 6.

### 5.2 GAE-DCSSNN component ablation

GAE and Swish both contribute to overall accuracy. Removing GAE drops accuracy by 1.56 pp, removing Swish drops it by 0.78 pp, and removing both drops it by 2.72 pp. Table 7 evaluates the GAE-DCSSNN component ablation results.

**Table 7:** GAE-DCSSNN component ablation, averaged across TOHWR and THCCMD.

Model	Acc. (%)	Prec. (%)	Rec. (%)	F-Meas. (%)
GAE-DCSSNN (full)	97.920	97.615	97.385	97.50
DCSSNN (no GAE)	96.360	95.930	95.715	95.82
GAE-DCNN (no Swish)	97.140	96.715	96.555	96.63
Plain DCNN (w/o GAE + Swish)	95.200	94.725	94.525	94.62

### 5.3 BEMFFIS membership function comparison

The BEMF yields the highest accuracy and lowest CER with competitive fuzzification and defuzzification time. Singleton and Triangular MFs offer lower computational cost but achieve lower accuracies of 93.455% and 94.975%, and higher CERs of 3.535% and 2.725%, respectively. Figure 4 shows accuracy and CER across all five variants, and Table 4 provides the corresponding timing values.

**Figure 4:**
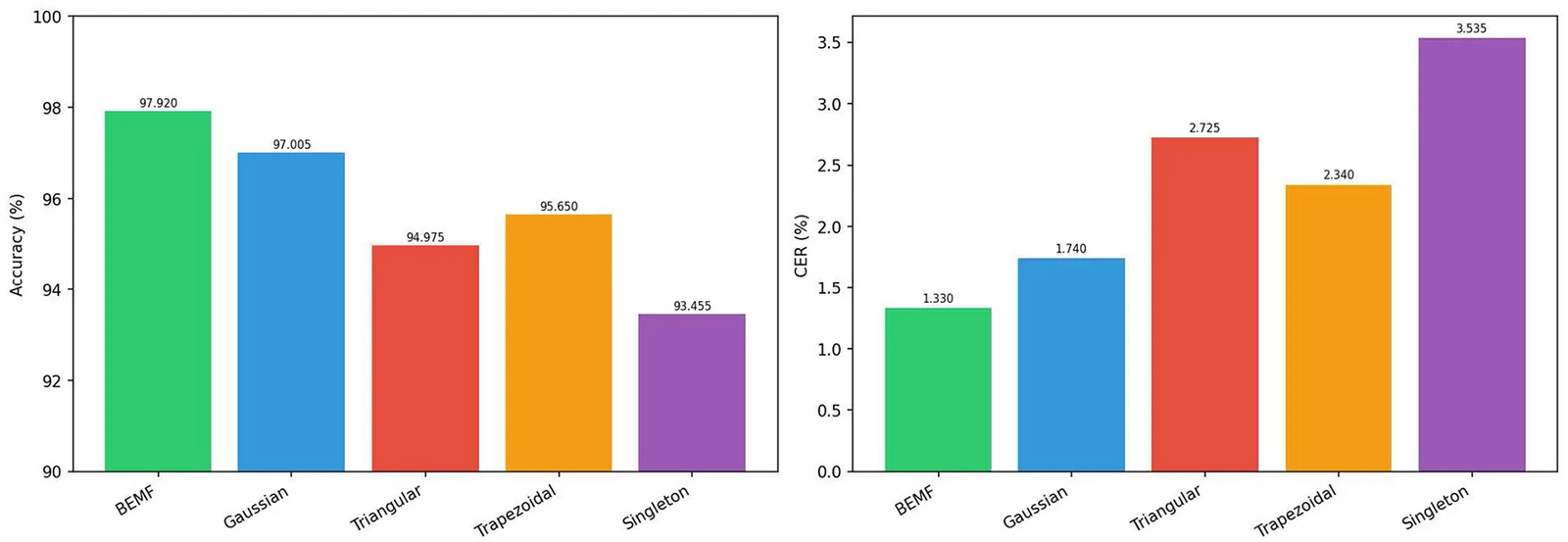
FIS membership function comparison across five variants. **Left**: accuracy, **Right**: CER. The BEMF indicates the best trade-off between accuracy and CER among the evaluated options. Fuzzification and defuzzification timing for each variant are reported in Table 4.

All metrics are reported on the 16,184-sample test partition. The five-baseline averages are: Acc. 94.37%, Prec. 93.89%, Rec. 93.59%, F-Meas. 93.73%, CER 3.57%.

### 5.4 mBBDART ablation

Removing DP reduces BLEU from 0.438 to 0.391 and METEOR from 0.512 to 0.473, indicating that syntactic structure aids translation quality. The mBART backbone without BD pruning scores a BLEU of 0.312. Adding BD pruning and DP improves this to a BLEU of 0.438, representing a relative gain of 40.4%. Figure 5 presents the full comparison against base transformer models. Detailed ablation metrics for mBBDART are provided in Table 8.

**Figure 5:**
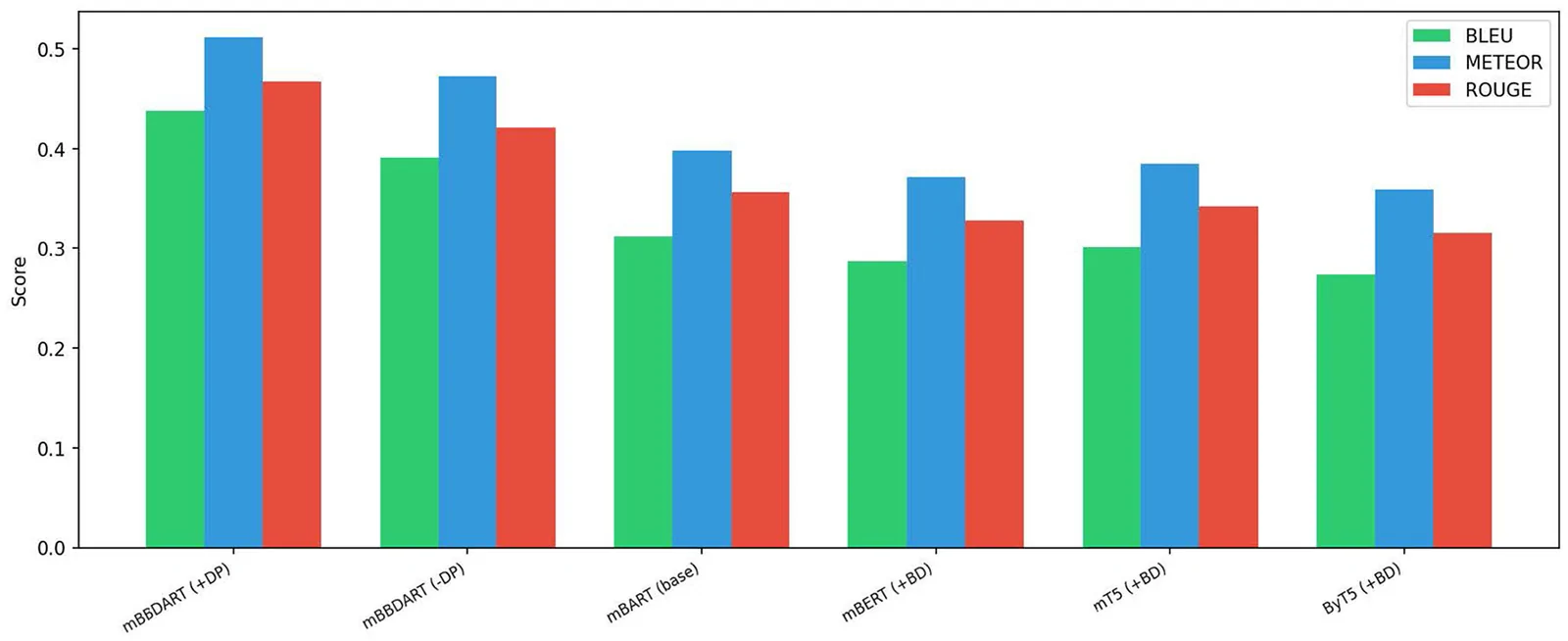
mBBDART vs. alternative transformer backbones on the 1,200-sample parallel annotation set. mBERT, mT5, and ByT5 are each augmented with the same BD pruning scheme as mBBDART. Base model averages: BLEU 0.294, METEOR 0.378, ROUGE 0.335. The full ablation detail is reported in Table 8.

**Table 8:** mBBDART Ablation on the 1,200-sample parallel annotation set.

Model	BLEU	METEOR	ROUGE
Proposed mBBDART (with DP)	0.438	0.512	0.467
mBBDART without DP	0.391	0.473	0.421
mBART (no BD, no DP)	0.312	0.398	0.356
mBERT with BD	0.287	0.371	0.328
mT5 with BD	0.301	0.385	0.342
ByT5 with BD	0.274	0.359	0.315

The proposed mBBDART is compared against its own ablation variants and against mBERT, mT5, and ByT5, each augmented with the same BD pruning scheme, to isolate the effect of the mBART backbone and DP.

## 6 Results and discussion

### 6.1 Qualitative pipeline visualization

Table 9 presents four representative samples from the test partitions, illustrating the visual output at each pipeline stage. The BEMFFIS and stroke extraction columns show structurally normalized skeletons from which GAE-DCSSNN extracts features. To quantify the impact of pre-processing, Peak Signal-to-Noise Ratio (PSNR) and Structural Similarity Index (SSIM) were computed between consecutive stages for the four sample images. The average PSNR between input and noise-removed images is 37.34 dB with an SSIM of 0.932, indicating that the median filter maintains structurally similar outputs while suppressing isolated high-frequency noise pixels. The average PSNR between input and contrast-enhanced images is 32.75 dB with an SSIM of 0.915, indicating that CLAHE introduces more significant pixel-level modifications while preserving structural content. The PSNR between noise-removed and contrast-enhanced images is 34.19 dB with an SSIM of 0.981. The multilingual output columns demonstrate Tamil-to-English and Tamil-to-Chinese translation via the mBBDART backbone.

**Table 9:** Stepwise qualitative visualization of the THCRT pipeline on four representative test samples.

Input	Noise removal	Contrast enh.	Char. seg.	Stroke ext.	Multilingual output	
					English	Chinese
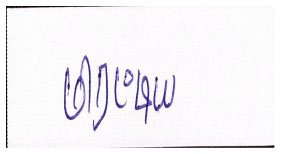	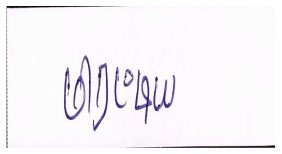	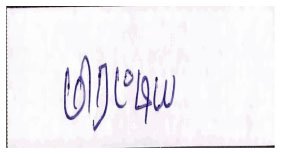	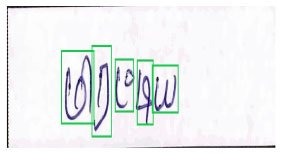	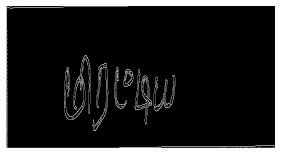	Threatened	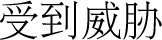
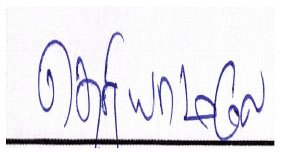	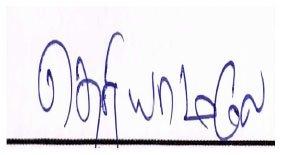	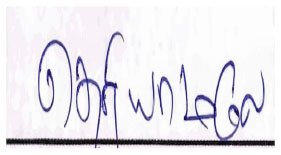	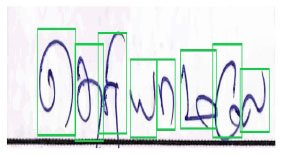	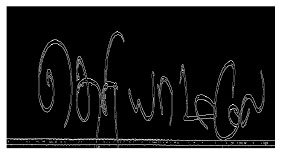	Unknowingly	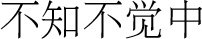
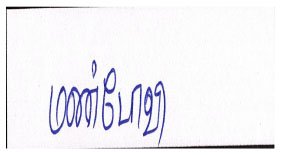	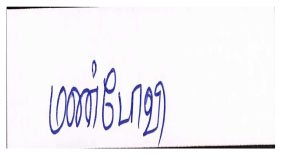	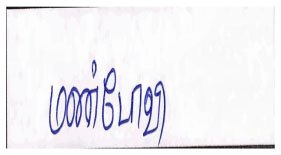	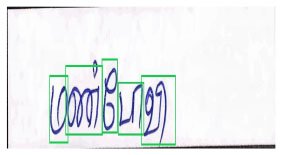	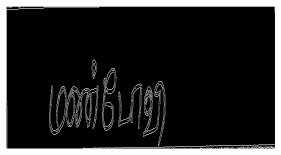	Mandovi	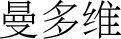
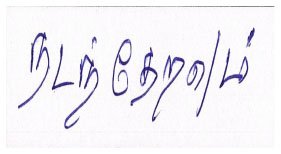	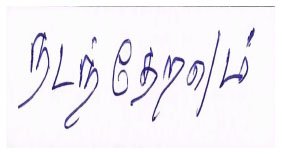	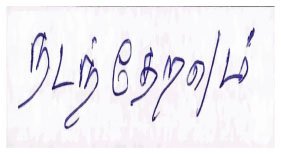	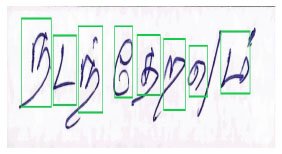	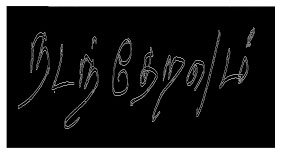	Conducted exams	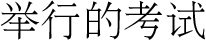

Each column shows the output of one pipeline stage. The character segmentation column allows visual comparison against the input column to assess CPFCA's boundary evaluation. The multilingual output demonstrates translation to English and Chinese via the mBBDART backbone. PSNR and SSIM values between consecutive stages are discussed in the text.

### 6.2 Recognition performance on TOHWR

The proposed GAE-DCSSNN achieves an accuracy of 98.23%, a precision of 97.85%, a recall of 97.62%, an F-measure of 97.73%, a CER of 1.12%, and an FNR of 0.0238 on the TOHWR test set. The total training time was 17,100 s at 342 s per epoch. Among the baselines, ResNet-50 yields an accuracy of 96.41% with an FNR of 0.0428, serving as a strong CNN reference. The five-baseline average FNR is 0.0641. The performance of the proposed model relates to two factors: BEMFFIS normalizes decorative variance before feature extraction, and the GAE excitation mechanism assigns higher relative weight to discriminative channels.

Figure 6 presents precision, recall, and F-measure for all models as a complement to Table 10. Figure 7 shows FNR across all models.

**Figure 6:**
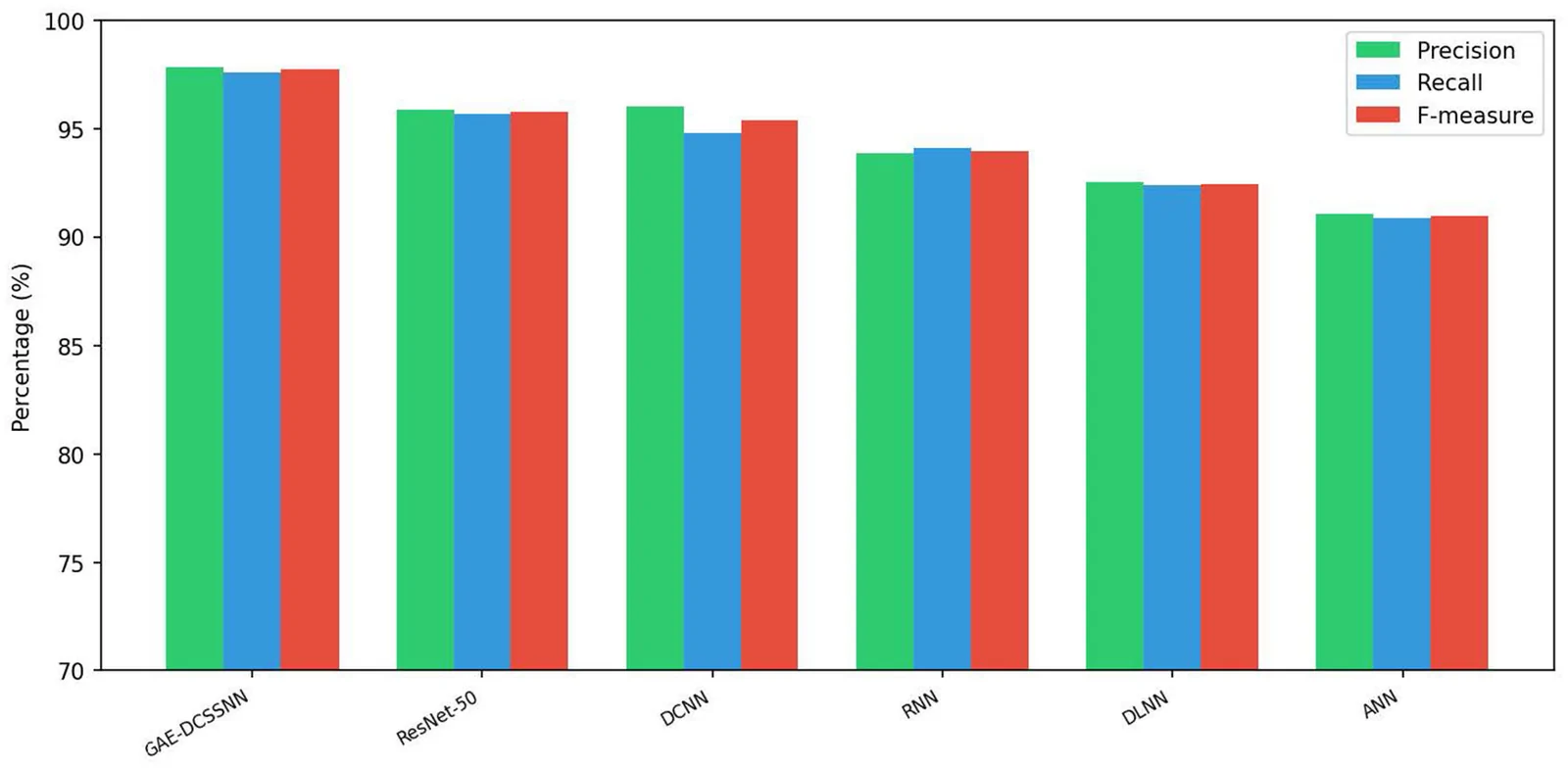
Precision, recall, and F-measure for all models on the TOHWR dataset, providing complementary analysis to Table 10.

**Table 10:** Comparative analysis on the TOHWR dataset.

Method	Acc. (%)	Prec. (%)	Rec. (%)	F-Meas. (%)	CER (%)
GAE-DCSSNN (Proposed)	98.23	97.85	97.62	97.73	1.12
ResNet-50	96.41	95.89	95.72	95.80	2.18
DCNN	95.87	96.04	94.83	95.43	2.65
RNN	94.53	93.91	94.12	94.01	3.42
DLNN	93.26	92.58	92.41	92.49	4.31
ANN	91.78	91.03	90.85	90.94	5.28

**Figure 7:**
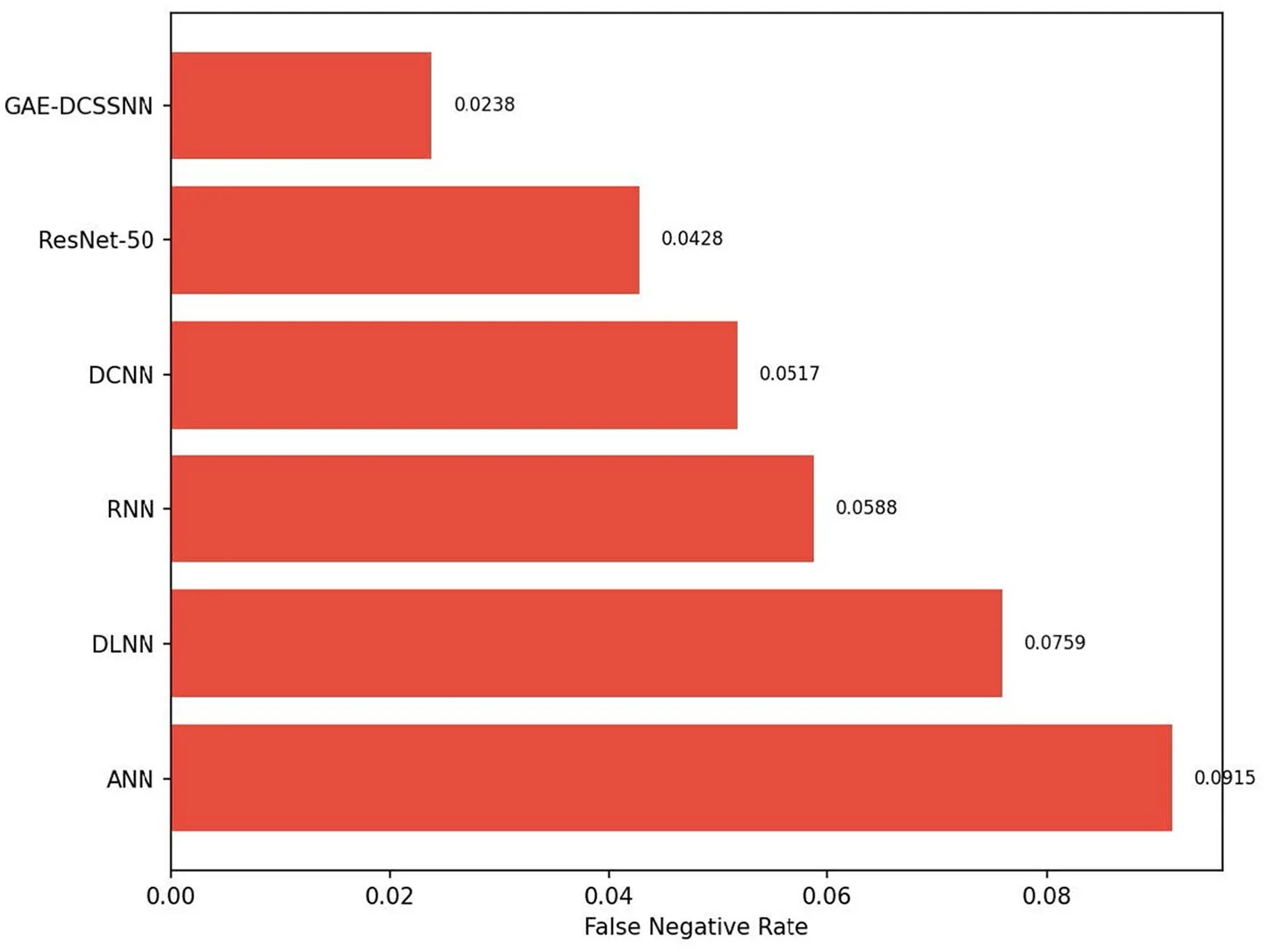
False negative rate, calculated as 1 − Recall, on the TOHWR dataset. Five-baseline average FNR: 0.0641. The proposed model yields an FNR of 0.0238.

### 6.3 Segmentation performance

CPFCA achieves an SR of 98.7%, an IoU of 96.3, and an ST of 12.4 ms per image. Figure 8 presents SR vs. IoU with bubble size proportional to ST, indicating that CPFCA maximizes SR and IoU while minimizing processing time. The advantage over CCA, which has an IoU of 91.8, is attributed to the Polar transformation linearising curved Dravidian baselines before component analysis. Table 11 summarizes the detailed segmentation performance metrics.

**Figure 8:**
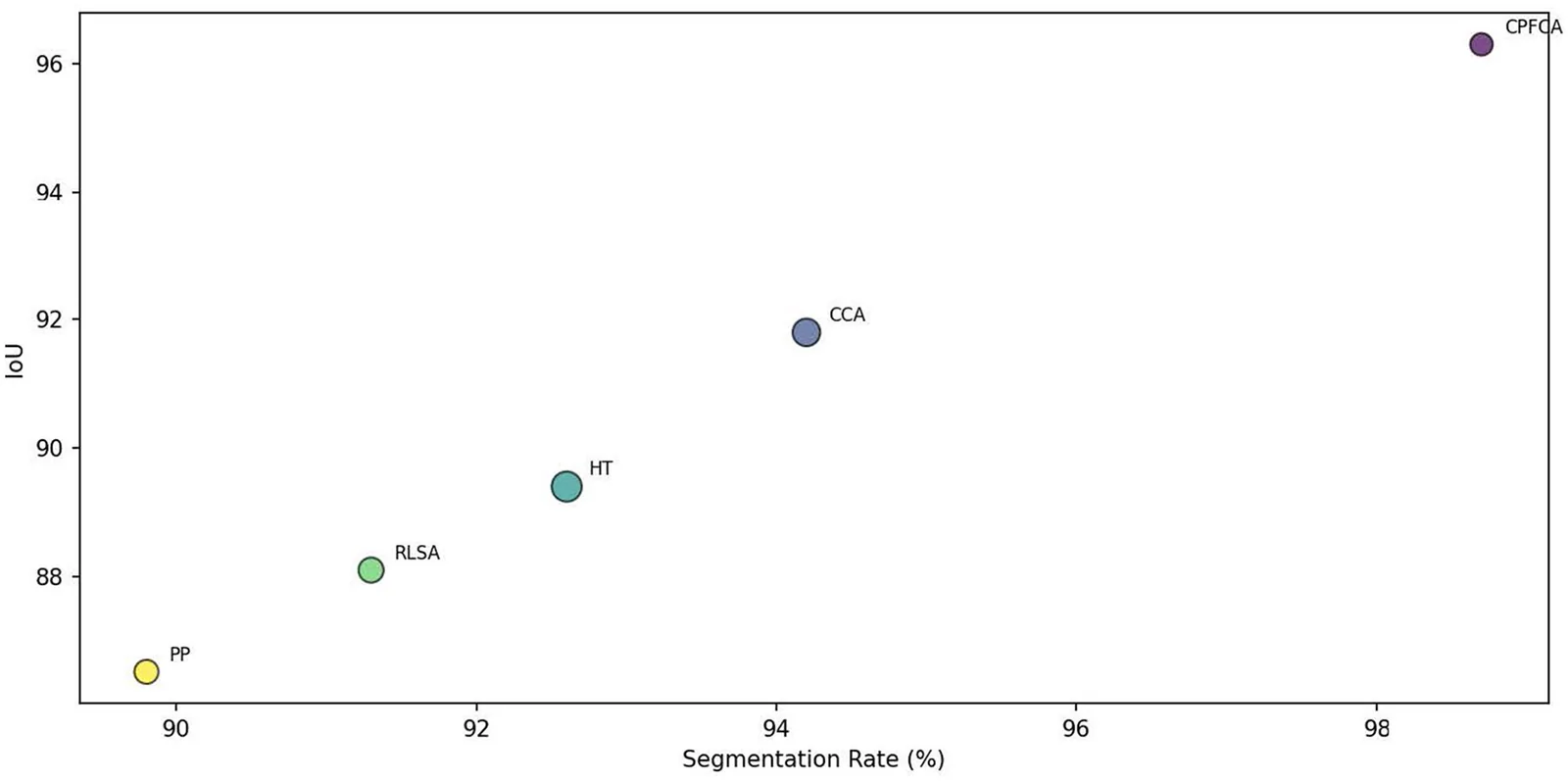
Segmentation performance as SR vs. IoU scatter plot, with bubble size proportional to segmentation time. CPFCA indicates a favorable trade-off among the three metrics.

**Table 11:** Segmentation performance.

Technique	SR (%)	IoU	ST (ms)
Proposed CPFCA	98.7	96.3	12.4
CCA	94.2	91.8	18.7
HT	92.6	89.4	22.3
RLSA	91.3	88.1	15.6
PP	89.8	86.5	14.2

ST is per-image segmentation time in milliseconds.

### 6.4 Recognition performance on THCCMD

On THCCMD, GAE-DCSSNN achieves an accuracy of 97.61%, a CER of 1.54%, and an F-measure of 97.26%. The average CER across both datasets is 1.330%, matching the ablation-averaged CER in Table 7. DCNN achieves the highest baseline accuracy at 95.93% on THCCMD, followed by ResNet-50 at 95.68%.

### 6.5 Comparison with state-of-the-art

The proposed THCRT framework achieves 98.23% on the quality-verified TOHWR benchmark. While this exceeds the 97.81% accuracy reported by Karthikeyan et al. (2023), it should be noted that their evaluation was conducted on a custom dataset rather than the standard TOHWR benchmark, making direct numerical comparison indicative rather than definitive. To establish a baseline for future comparisons under equivalent evaluation conditions, it is noted that the THCRT framework achieves 96.14% on the original, unverified TOHWR distribution, as detailed in Table 12. This indicates that the maximum attainable accuracy is closely constrained by ground-truth label quality. Maheswari et al. (2024) reports 96.04% but evaluates on ancient palm leaf manuscripts with a different data distribution where the absence of fuzzy pre-processing limits generalization to variable handwriting styles. Devendiran et al. (2024) evaluated VGG-19 on a custom dataset, reaching 98.34% accuracy, but lacks a curved-baseline segmentation mechanism analogous to CPFCA.

**Table 12:** Recognition accuracy on unverified vs. quality-verified benchmark partitions.

Partition	Accuracy (%)	
	TOHWR	THCCMD
Unverified benchmark	96.14	95.31
Quality-verified benchmark	98.23	97.61

The model is trained and evaluated identically in both conditions. The difference reflects benchmark label quality.

Furthermore, few prior works in the compared literature have evaluated their architectures on the comprehensive 247-class THCCMD dataset. Ravi (2024) evaluated VGG-16 on a restricted custom dataset, yielding 71.90% accuracy. Both Anbalagan et al. (2026) and Rastogi et al. (2026) are cited for their architectural contributions in Section 2, but their results on their respective benchmarks are not directly comparable to the THCCMD performance. Table 13 presents a comparison of the THCRT framework against existing works.

**Table 13:** Comparison against existing works in Tamil handwritten character recognition.

Study	Method	Acc. (%)	Prec. (%)	Rec. (%)
Proposed THCRT	GAE-DCSSNN + BEMFFIS + mBBDART	98.23	97.85	97.62
Karthikeyan et al. (2023)	SLOA-TL + Inception V3	97.81	—	95.0
Devendiran et al. (2024)	VGG-19	98.34	—	—
Maheswari et al. (2024)	CNN	96.04	—	—
Ravi (2024)	VGG-16	71.90	—	—

Baseline accuracy metrics denote peak performance reported by authors on their respective custom or proprietary datasets, whereas the proposed THCRT model is evaluated on the standard TOHWR benchmark. Direct numerical comparison is therefore indicative rather than definitive.

### 6.6 Quality verification impact analysis

Quality verification accuracy was assessed across six architectures to evaluate the effect of label correction. Table 12 reports per-architecture improvements on the TOHWR benchmark: GAE-DCSSNN +2.09, ResNet-50 +2.78, DCNN +1.43, RNN +2.34, DLNN +1.67, ANN +2.51 pp. The mean improvement across all six architectures is 2.14 pp, with a range of 1.43–2.78 and a standard deviation of 0.51. Figure 9 presents the comparison.

**Figure 9:**
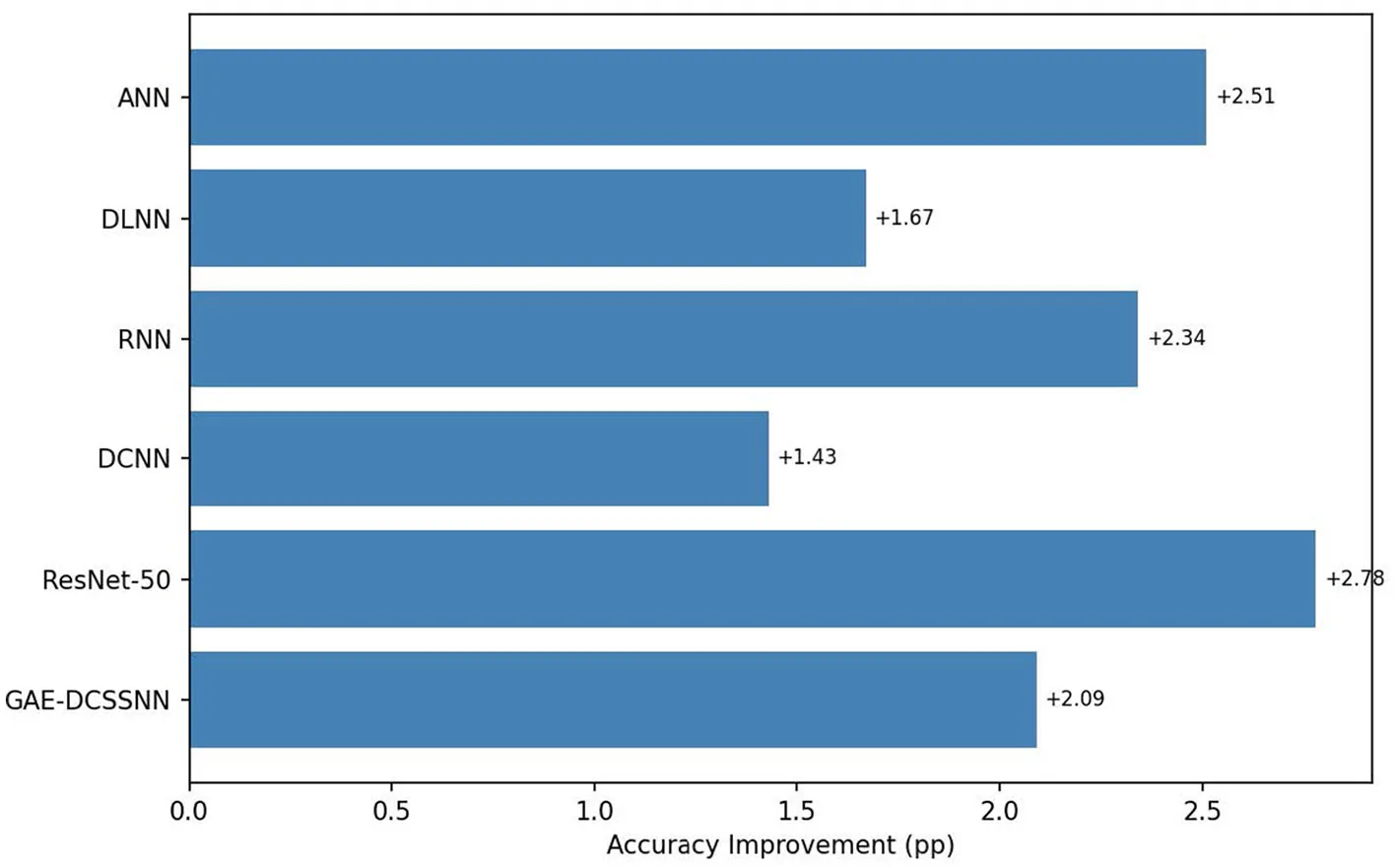
Accuracy improvement after quality verification across six architectures on the TOHWR benchmark. Mean improvement: +2.14 pp, range 1.43–2.78. All values represent TOHWR test-set accuracy.

### 6.7 Cross-validation

The minimal and non-monotonic fold-to-fold variation in both datasets indicates model stability and mitigates ordered-split artifacts, as shown in Figure 10.

**Figure 10:**
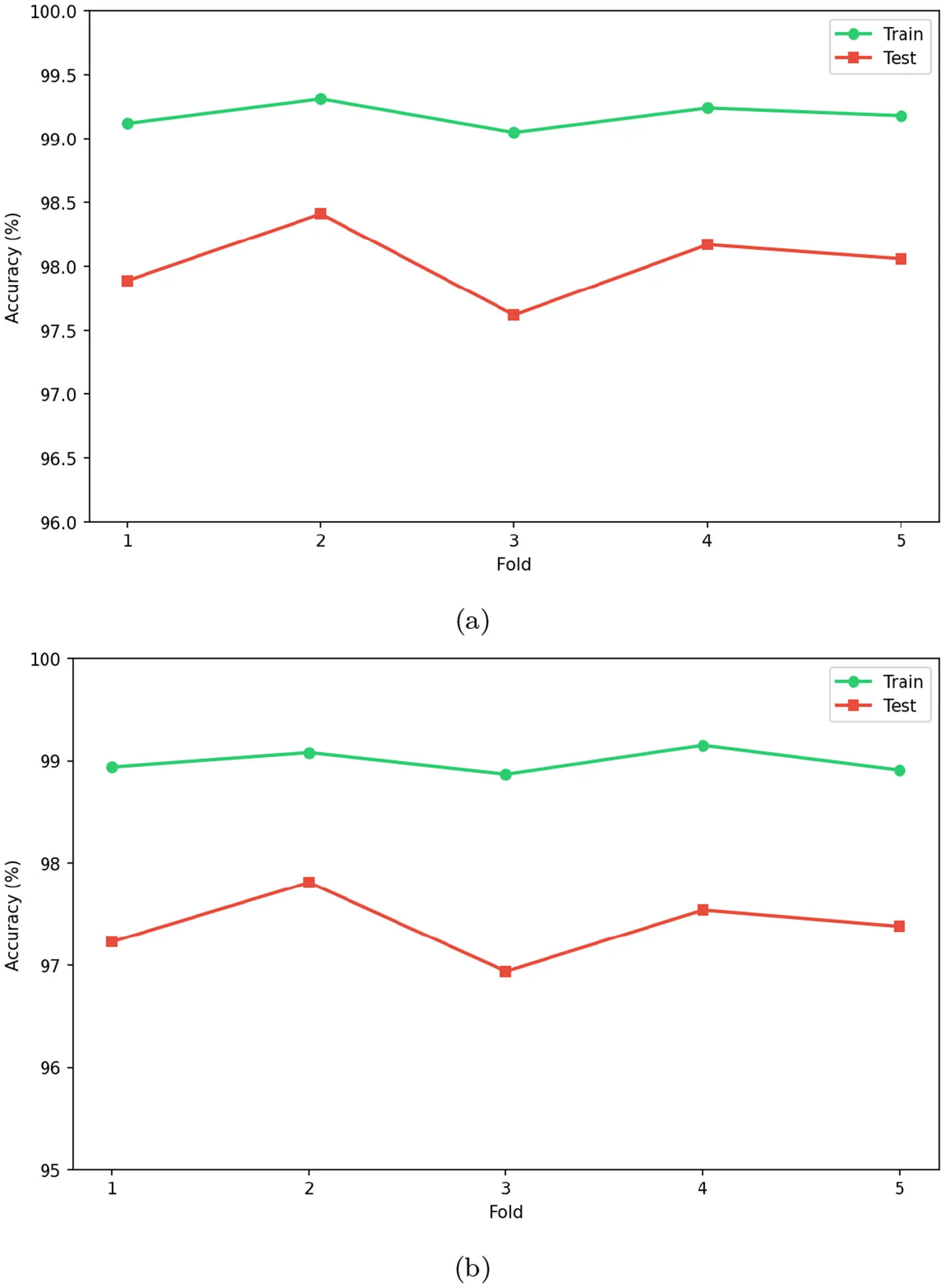
Five-fold cross-validation accuracy for **(a)** TOHWR and **(b)** THCCMD using random stratified splits. TOHWR: training 99.05–99.31%, testing 97.62–98.41%. THCCMD: training 98.87–99.15%, testing 96.94–97.81%.

### 6.8 Ground truth quality verification

4A data quality assurance step was conducted prior to finalizing train/test splits to identify and correct mislabelled or ambiguous ground truth entries in the original dataset releases. Two annotators with Cohen's κ = 0.87 reviewed samples for visual confusions and contextual misinterpretations. Table 12 shows accuracy on the unverified benchmark compared to the quality-verified partition, and Figure 11 shows the corresponding CER and error rate reductions. The model is trained and evaluated identically in both conditions. All primary results throughout this paper are reported on the quality-verified benchmark.

**Figure 11:**
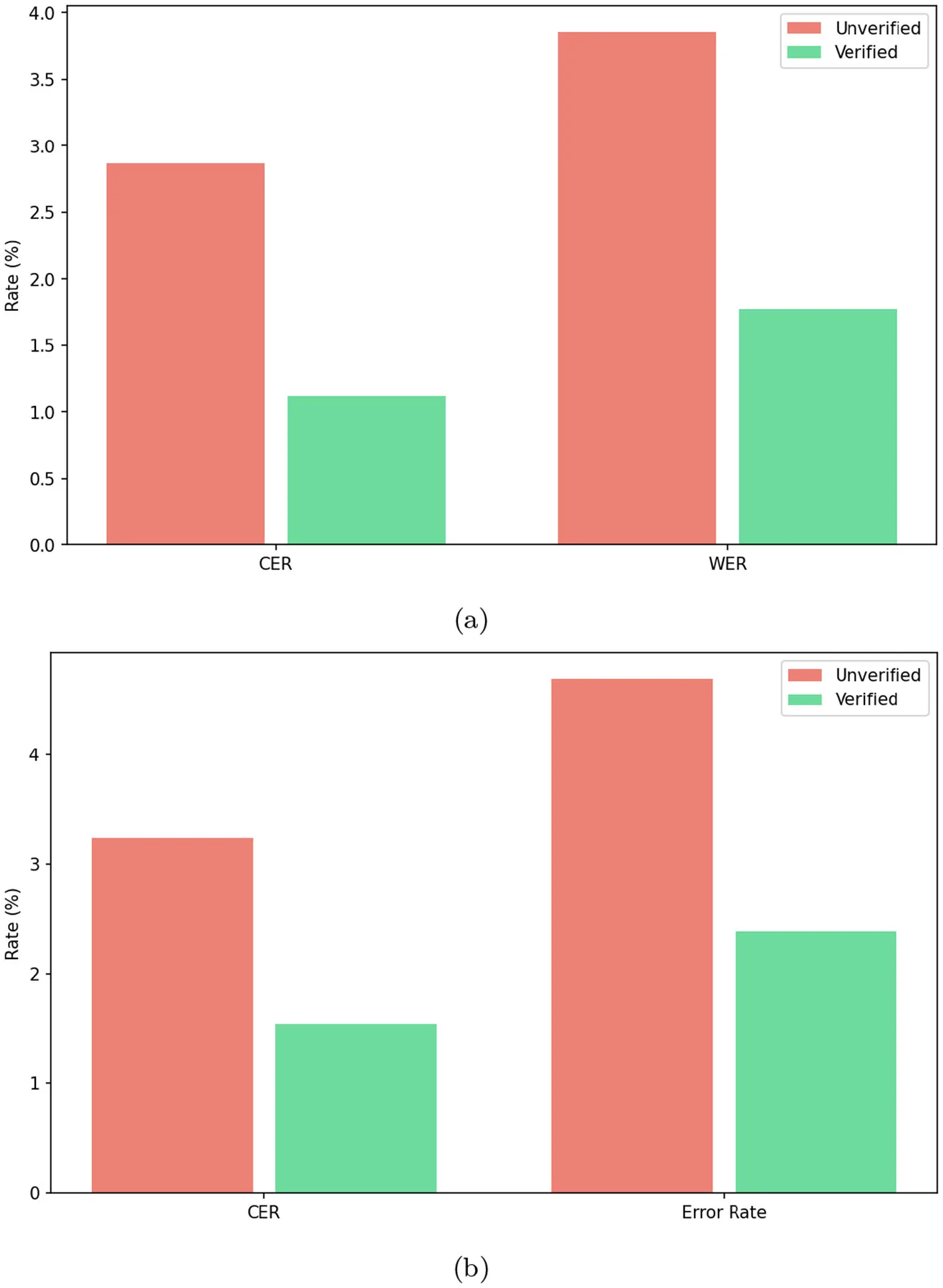
CER and error rate on unverified vs. quality-verified partitions for **(a)** TOHWR: CER 2.87% → 1.12%, WER 3.86% → 1.77%; **(b)** THCCMD: CER 3.24% → 1.54%, Error Rate 4.69% → 2.39%.

### 6.9 Confusion matrix analysis

Figure 12 presents the classification outcome summary. On TOHWR, the THCRT framework correctly classified 15,898 out of 16,184 test samples, achieving 98.23% accuracy. On THCCMD, 4,822 out of 4,940 test samples were correctly classified, achieving 97.61% accuracy. As reported in Table 14, the precision of 97.38%, recall of 97.15%, and F-measure of 97.26% closely track overall accuracy, indicating that performance is not driven by a small number of dominant classes.

**Figure 12:**
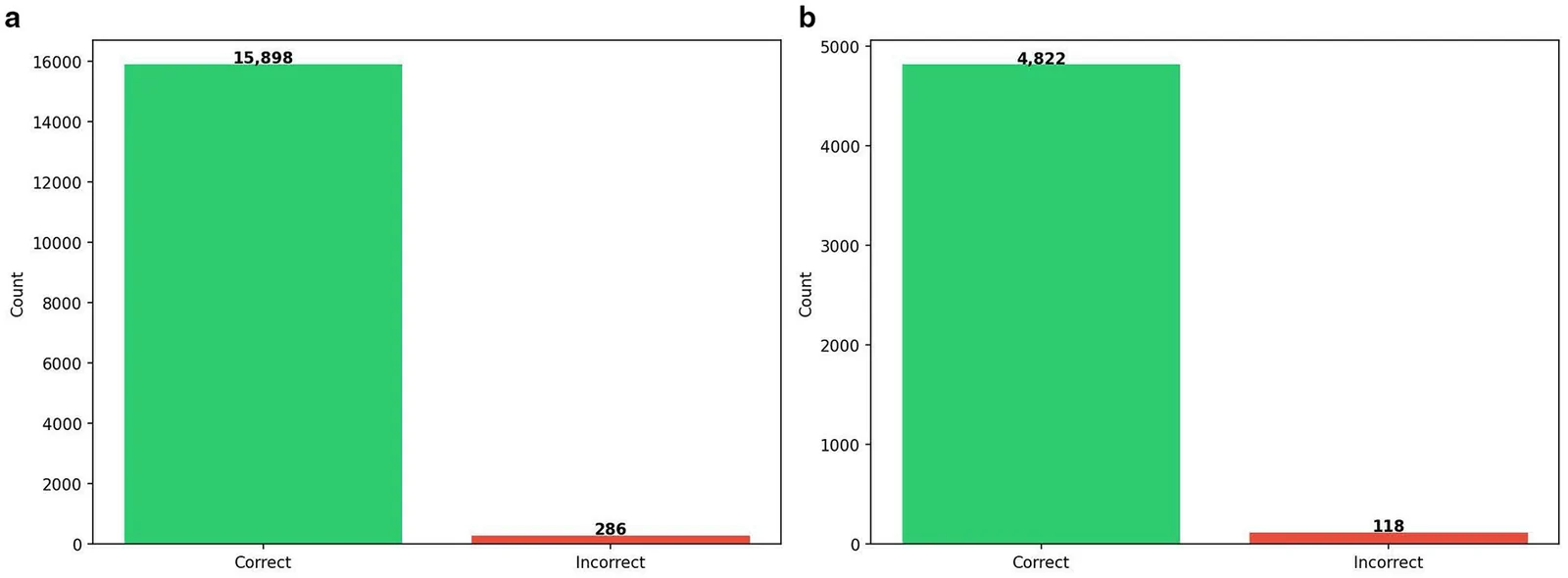
Classification outcome summary for **(a)** TOHWR and **(b)** THCCMD. TOHWR: 15,898 correct and 286 incorrect out of 16,184 samples, yielding accuracy 98.23%. THCCMD: 4,822 correct and 118 incorrect out of 4,940 samples, yielding accuracy 97.61%. A full class-level confusion matrix is impractical to print for either dataset with 13,294 classes for TOHWR and 247 for THCCMD. The precision, recall, and F-measure in Table 14 provide class-level performance summaries.

**Table 14:** Comparison on the THCCMD dataset, comprising 247 character classes and 4,940 test samples.

Method	Acc. (%)	Prec. (%)	Rec. (%)	F-Meas. (%)	CER (%)
GAE-DCSSNN (Proposed)	97.61	97.38	97.15	97.26	1.54
DCNN	95.93	95.51	95.38	95.44	2.34
ResNet-50	95.68	95.74	94.82	95.28	2.51
RNN	93.82	93.19	93.04	93.11	3.95
ANN	92.45	91.73	92.14	91.93	4.89
DLNN	91.37	91.92	90.48	91.19	5.67

CER is added to enable direct comparison with Table 10. The five-baseline averages are: Acc. 93.85%, CER 3.87%. Note on CER for THCCMD: CER is computed as the Levenshtein distance between the predicted and true character's Unicode codepoint representation, normalized by reference length. The CER of 1.54% is lower than the classification error rate of 2.39% because misclassifications tend to involve characters differing by a single diacritical codepoint. CER is included to enable direct numerical comparison with the TOHWR CER in Table 10.

To characterize class-specific recognition behavior on the 247-class THCCMD dataset, per-class accuracy was computed across all classes utilizing 20 test samples per class. Mean per-class accuracy is 97.61%, identical to overall accuracy as expected under the class-balanced test partition, with a standard deviation of 3.24% and a range of 80.00% to 100.00%. Approximately 12 of the 247 classes fall below 95% per-class accuracy. The lowest-performing class, operating at 80.00% accuracy with 4 of 20 samples misclassified, accounts for the single most frequent confusion pair observed in the dataset, in which all four of its errors are attributed to one visually similar adjacent class. This concentration of errors within a small number of confusable class pairs, rather than uniform degradation across all classes, is consistent with the character-level visual similarities characteristic of Tamil script.

## 7 Conclusion

This paper presented the THCRT framework, a fuzzy-augmented deep learning NLP pipeline for multilingual translation from handwritten Tamil script. The BEMFFIS module uses a Biparametric Exponential Membership Function to model stroke-boundary uncertainty between segmentation and feature extraction. A pipeline ablation study showed that BEMFFIS yields a 1.79 pp accuracy gain and a 1.49 × CER increase when removed relative to the full pipeline. Replacing CPFCA with standard CCA reduces accuracy by a further 0.955 pp, demonstrating the contribution of curved-baseline segmentation. Removing DP from mBBDART reduces BLEU by 0.047, highlighting the role of syntactic structure in translation quality.

The THCRT framework achieves an accuracy of 98.23% and a CER of 1.12% on TOHWR with an item error rate of 1.77% and a WER of 1.77%, and an accuracy of 97.61% on THCCMD with a CER of 1.54% and an error rate of 2.39%, outperforming the compared baselines on both datasets. The mBBDART translation model yields a BLEU of 0.438 and a METEOR of 0.512 on the 1,200-sample parallel annotation set with Cohen's κ = 0.91.

### 7.1 Limitations and future scope

The THCRT framework does not explicitly model inter-writer stylistic variability and assumes consistent writing style within a document. The recognition component is Tamil-specific, and extension to Telugu, Kannada, and other Dravidian scripts is a planned future direction. Translation metrics are evaluated on a constrained short-phrase vocabulary. Document-level evaluation on FLORES or Samanantar remains future work. Future work will also address style-transfer adaptation for diverse handwriting and end-to-end differentiable architectures to minimize error propagation across pipeline stages.

## Data Availability

The datasets used are publicly available at https://www.kaggle.com/datasets/sabarinathan/tamil-offline-handwritten-word-recognition/data for the TOHWR dataset, https://data.mendeley.com/datasets/6zcpgchvmx/1 for the THCCMD dataset, and https://huggingface.co/datasets/harsha360/thcr-parallel-1200 for the 1,200-item parallel annotation dataset.
